# A Treatise on Neuralgia

**Published:** 1839-04

**Authors:** 


Art. V.—*A Treatise on Neuralgia.* By Richard Rowland, m.d.—*London*, 1838. 8vo. pp. 173.

There is a peculiar unsatisfactoriness in the term neuralgia, as it is applied to designate a symptom of very various diseases. A tumour presses on a nerve, or a nerve is so complicated in the growth of a tumour as to give rise to extreme pain; the cause consists in accumulated fæces, diseased bone, organic disease within the head of various kind, carious teeth, acid secretions in the stomach, &c.; and we call a peculiar pain produced by these and other concurrent causes, neuralgia. The volume before us is a creditable monument of its author's industry in collecting together multitudinous forms of neuralgia from very various sources; but the very nature of the subject is such, unfortunately, that, after having perused the book, we feel the need of some knowledge by which to arrange and systematize the various facts, so as to deduce from them some sound pathological principles and some definite and satisfactory rules of practice; and for this purpose a large number of present facts require, from their incompleteness, to be entirely rejected; they are useless as affording ground for any legitimate deductions. We do not mean by these observations to condemn Dr. Rowland's book, which is well worthy of notice.

The volume commences with some general considerations on the subject of neuralgia; particular forms of the disease are next treated of as occurring in external nerves; the consideration of the disease as occupying internal organs, together with some cases, terminating the volume.

We shall allude to Dr. Rowland's observations on spinal tenderness as accompanying various forms of neuralgia; a subject which we believe to be as much overrated by some medical men as it is underrated by others. We do not mean to advocate any theory on the subject; but merely to state as a fact, that there exist certain pains of a neuralgic character; that tenderness on pressure of the spinous processes of some one or more vertebræ coexists with such pains; and that relief is obtained by remedies applied to such painful vertebræ. The frequency of such a condition, we believe, has been much exaggerated; and we are glad to find Dr. Rowland stating that, "with regard to the frequency of neuralgia from spinal irritation, my opinion does not coincide with the opinion of some recent authors; for, in the majority of cases, I have not been able to detect any sign of tenderness over the vertebral column: I have the authority of Dr. Alison for saying that he has arrived at the same conclusion." It is a question in these cases how far the spinal tenderness is a mere associate of the other pains; for it is not unfrequently found that a tenderness, apparently of the same character, exists in individuals apparently in good health. The same sign has been much lauded of late as a method of distinguishing from inflammation such abdominal pains as are of a neuralgic character. On this point Dr. Rowland speaks satisfactorily from his own experience. "After a careful examination of a large number of cases, I feel justified in remarking that this diagnostic sign cannot be trusted with safety. It is entirely absent in many cases of visceral neuralgia: and, in some cases of chronic visceral inflammation, unattended with disease of the spine, tenderness over the vertebral column is present." We think it worth noticing that the author has found that the troublesome pains which sometimes accompany shingles, are often instantaneously relieved by touching the painful vesicles with lunar caustic: but that it is generally better to apply the caustic to the whole of each group, or to cover them with a strong solution of this substance. In that affection termed by Sir A. Cooper *irritable breast*, (by the author, *neuralgia mammæ*,) "much benefit may be derived, at least in the milder form of this affection, by the application of leeches, or by cupping over the sacrum or in the uterine region. I have often," says Dr. Rowland, "seen the symptoms disappear under this treatment, when no application has been made to the breast." It does not consist with our limits to make any further extracts from the work before us, which will, however, repay the trouble of an attentive perusal.

